# Lipomas in Patients of Gynecomastia: Is It of Any Relevance?

**DOI:** 10.1055/s-0045-1802630

**Published:** 2025-02-05

**Authors:** Sudhanshu Punia, Aakanksha Goel, Amit Gupta

**Affiliations:** 1 Divine Cosmetic Surgery, Greater Kailash, New Delhi, India

Gynecomastia is the most common benign condition affecting the male breast. 1 Lipoma is the commonest benign soft tissue neoplasm of mature adipose tissue. 2 Gynecomastia is the most common cause of male breast enlargement, 3 whereas lipoma is the second most common cause. Lipomas occur in all parts of the body and are mostly small and 20% occur in the chest wall. 4 Many patients can have generalized lipomas, which may be numerous and/or show bilateral distribution. 5 As it is termed “universal tumor,” it can occur at any anatomical site where fat is present; however, it is less commonly reported in the male breast. Coexistence of both pathologies is rare, but given how common they are, their presence together might be more common than believed. Lipomas are usually treated with direct excision, but newer modalities include suction-assisted liposuction (SAL), 6 intralesional deoxycholic acid injections, focused ultrasound, and 1,444-nm lasers, but as SAL is the most common available modality and is already being used at the time of gynecomastia surgery, it helps to address lipomas as well.

Most patients gave no prior history of lipomas, whereas some reported lipomas elsewhere. About 50 to 60 gynecomastia patients are operated monthly, out of which 10 to 12 patients are found to have lipomas (at the time of surgery), more commonly in generally fatty patients or those with a higher gynecomastia grade. When lipomas are noted during the initial examination or reported by the patient themself elsewhere, extra care is taken to identify lumps in the chest while doing liposuction and to break them by suction. We also meticulously look for the presence of any bleeders. If lipomas are found directly at the time of surgery, care is taken to suction the lipomas without causing any contour abnormalities overall while taking care of the previous points. The residual gland is firmer in touch and lipomas are softer, move, and have a characteristic yellow color when visible through the incisions, which aid in their removal. As patients do not have any lumps elsewhere or do not report their presence, the majority (>95%) are discovered intraoperatively.

We noted the following:

- Lipomas become evident after liposuction.
- Lipomas are more commonly found near the nipple–areola complex (NAC).
- Lipomas get excised with gland removal.
- Typically, such patients bleed more than usual.
- If missed, lipomas can cause visible and palpable lumps, which patients are concerned about.

Gynecomastia has a major aesthetic component; removal of any lump-like lipoma, even if asymptomatic, should be done, as a visible chest lump after surgery would be counterproductive, resulting in patient dissatisfaction. We feel that when the surgeon encounters these, all attempts must be made to remove the lipomas and cauterize the feeder vessels. Feeder vessels can be a source of troublesome bleeding, necessitating meticulous hemostasis or drain placement, which normally is not done, as vessels may cause unnecessary vexing bleeding or hematomas if left uncontrolled, resulting in an avoidable complication. In case removal would result in a depression or the lipoma is peripheral, it should be broken with a basket cannula or if it is near the NAC, it should be leveled out by a pair of tissue cutting scissors.

Patients having multiple lipomas over their bodies can have them on their chest and may be unaware about their presence. When encountered during treatment for gynecomastia, the lipomas should be removed and meticulous hemostasis should be achieved to ensure optimal results and avoid complications, making them a relevant finding that should be dealt with. Residual lumps and unexplained bleeding in otherwise normal gynecomastia patients may be attributed to lipomas.
